# Does Digitization Promote Green Innovation? Evidence from China

**DOI:** 10.3390/ijerph20053893

**Published:** 2023-02-22

**Authors:** Chuanlin Wang, Guowan Yan, Juan Ou

**Affiliations:** 1 School of Economics and Business Administration, Chongqing University, Chongqing 400044, China; 2 International Business School, University of International Business and Economics, Beijing 100105, China

**Keywords:** enterprise digitization, green innovation, resource allocation, cleaner production

## Abstract

Green innovation is an important strategy in seeking sustainable competitive advantages. This paper investigates the impact of enterprise digitization on green innovation and its mechanisms. We find that enterprise digital transformation has a significant effect on the promotion of green innovation. This positive effect mainly stems from the advantage of resource reallocation generated by enterprise digitalization that can alleviate financing constraints and raise risk-taking levels. Furthermore, the level of economic development strengthens the impact of enterprise digitization on green innovation, and the positive relationship between enterprise digitization and green innovation is stronger in regions with stronger environmental regulation and higher intellectual property protection, as well as in state-owned and heavily polluting enterprises. Digitization can optimize resource utilization, strengthen the capacity of green innovation in pollution reduction and promote the clean production of enterprises. Our results show that enterprise digitization plays a positive role in innovation activities. Furthermore, our results show that enterprise digitization plays a positive role in innovation activities.

## 1. Introduction

With the rapid growth of the global economy, consequent severe ecological issues, such as resource exhaustion and environmental pollution, have become major constraints to sustainable development [1]. As a vital market player in emerging economies, China will, as Chinese President Xi Jinping pointed out at the 75th UN General Assembly, spare no effort in taking more effective steps in pursuit of the commitments that its peak carbon dioxide emissions will be achieved by 2030 and that carbon neutrality will be achieved by 2060. In view of this, it is imperative to carry out green transformation for micro-businesses with more stringent environmental policies and measures [2] since they are not only the anchor of economic growth but are also direct polluters that produce the most pollution. Therefore, it is necessary to resort to green innovation, which is a crucial strategy for seeking sustainable competitive advantages [3,4], by applying technological creativity to ecological protection with the aim of effectively reducing resource consumption and environmental pollution and, thus, constantly yield economic benefits [5,6]. However, Chinese companies are faced with daunting challenges as one of the emerging economies that are often characterized by an imperfect market and an excessive pursuit of growth. With the recent development of digital technologies highlighted by Internet plus, artificial intelligence and big data, more and more businesses, in an effort to conduct digital transformation, expect information-technology-driven upgrades to sharpen their competitive advantages in the market. According to the SMARTer2030 report released by the Global Enabling Sustainability Initiative (GeSI), digital technologies have the potential to enable a 20% reduction in global CO₂ emissions by 2030 (Global Enabling Sustainability Initiative (GeSI: https://gesi.org/research/smarter2030-ict-solutions-for-21st-century-challenges accessed on 22 December 2022). As the commercial applications of digital technologies have gradually taken root, discussing the driving mechanism of corporate green innovation serves to be the micro-foundation for achieving objectives of environmental protection and environmental development.

Enterprise digitization refers to the comprehensive collaboration of products, services, processes, modes and organizations by combining digital technologies such as information, computing, communication and connectivity [7,8]. Digital technologies that lead to effective information flow and resource reallocation have a positive role in corporate governance [9], the information environment [10] and production efficiency [11] since production, management, organizational structure and the business model are fundamentally changed [12,13,14,15]. The existing literature has largely discussed the relationship between the digital economy and environmental protection from a macro perspective (e.g., ecosystem [16], energy consumption [17,18]) and how external digital environment influence companies’ green behaviors [19,20,21]. Digital transformation in the production and operation process of enterprises may promote green innovation in two aspects. First, digital technology can enable enterprises in a transformation in terms of structure, psychology and resources [22]. Enterprise digitization transforms the original production and operation mode by applying cutting-edge information and communication technology. This advanced digital communication technology, when put into use, contains the qualities of technological progress [23]. Second, digital transformation not only involves the comprehensive collaboration within the enterprise but also involves the external collaboration between the enterprise and the multi-subject [24]. Thus, the integration of digital technology and technological advances with other production factors can promote changes and effective connections in various aspects of enterprise production, supply chain and sales [25], which can more directly realize the optimal allocation of enterprise production factors, accelerate information transmission and improve the green innovation capability of enterprises.

Researchers in current studies focusing on the digitalization–green innovation relationship have not reached a consensus. On the one hand, digital transformation may have a positive impact on innovation by mitigating internal information asymmetry and improving the efficiency of information transmission [26,27]. On the other hand, although creativity information and communication are, by dint of digital technologies, no longer confined to space, the negative effects may be seen when innovation resources are squeezed and information is overloaded due to a limited information processing capacity [28,29]. This divergence is, from our point of view, majorly attributed to the limitations to which these studies were subject. First, these studies are based on location-specific data derived from varying economic environments and ecological regulations in the world. However, for businesses in emerging economies, it is the prevailing financial constraints [30] and the exclusive pursuit of economic interests that are the key factors stifling green innovation. Second, some studies investigated the two stands by detecting whether there is a correlation between corporate digitalization and green behaviors as well as the influence path. In those studies, a positive correlation indicates that the use of digital technologies with the aim of improving the information environment is a vital practice to realize businesses’ long-term strategies and enhance their green governance [26,27], while the negative one refers to the scenario wherein the investments in innovation may be squeezed due to the activities of digital transformation, thus influencing innovation efficiency [28,29], which is a conclusion based on an assumed trade-off relationship between digital transformation and green innovation, ignoring the fact that digitalization is a continuous process which may help reallocate business resources or introduce new resources [31,32]. In addition, there are endogenous uncertainties in the examination of such relationships. Firms’ green innovation may weigh on, in turn, deeper digitalization that constantly nurtures the former—a presumable reverse causality. At the same time, digital transformation may coincide with green innovation since both of them are the decisions made by the executives of a company. Therefore, on the basis of solving the endogenous problems of the model, it is necessary to explore whether and how to promote green innovation through enterprise digitalization in emerging markets and to provide theoretical support and a scientific basis for the coordinated development of digitalization and green innovation under the “double carbon” objective.

In this study, we use data from Chinese-listed companies to study the role of digitalization on green innovation and answer four levels of questions: (1) Do firms with higher levels of enterprise digitization have stronger levels of green innovation? (2) If there is a significant green innovation effect of enterprise digitization, what is the transmission mechanism of digitization affecting enterprise green innovation? (3) What are the effects of different external and internal conditions on the green innovation effect of digitalization? (4) Can green innovation characterized by digital technology achieve a cleaner production of enterprises? The empirical results show that enterprise digitalization significantly contributes to green innovation, showing that digital transformation has a significantly positive effect on green innovation by enhancing companies’ abilities to obtain funding resources and improving their risk tolerance. Our results continue to hold after addressing the endogenous problems through several approaches. Further, our results indicate that such a positive effect is intensified in regions with advanced economic development, strict environmental regulation and complete intellectual property protection, as well as in state-owned companies and high-polluting industries. Finally, we also find that enterprises can realize the environmental protection strategy by improving the efficiency of resource utilization and then achieve the goal of energy saving and cleaner production.

Our study may have three contributions. First, this paper enriches the conclusions about the relationship between digitalization and green innovation. There is no consistent conclusion on the relationship between digitalization and green innovation [26,27,28,29]. Although Jiang et al. [33] partially focus on the resource allocation effect using government subsidies and R&D investment indicators, this paper incorporates the behavioral patterns of enterprises shaped by digitalization and innovation resource allocation into the research framework of the relationship between enterprise digitalization and green innovation based on the behavioral characteristics of enterprises in emerging economies, reveals the inner logic of digitalization for green innovation from the perspectives of “financing constraints” and “risk taking” and deepens the understanding of the resource soil on which green innovation depends and the logic of its power. This provides explanatory ideas for the existing literature to address the differential findings of corporate digitalization affecting corporate green behavior.

Second, distinguishing it from the existing literature, this paper clarifies the mechanism of how the digital economy and green innovation work to influence each other from several perspectives. Although some studies have found that the level of regional economic development is an important factor affecting the green innovation of enterprises [34,35,36]. In the relationship between digitalization and the green innovation of enterprises, this paper further finds that the level of economic development strengthens the positive relationship between digitalization and green innovation, and the green innovation effect of enterprise digitalization mainly exists in enterprises in regions with stronger environmental regulation and higher intellectual property protection, as well as in state-owned and heavily polluting enterprises. It provides a new research direction for investigating the determinants of green innovation of enterprises, which may be applicable to emerging economies and developed countries.

Third, since the relevant studies have mainly examined the impact of digitalization limited to aspects such as corporate green behavior without paying the necessary attention to the latter’s value judgment [19,20,21,26,27]. This paper examines the impact of green innovation characterized by digital technology on cleaner production in enterprises to demonstrate the rationality and environmental importance of the positive correlation between digitalization and green innovation. This test is especially important for heavy polluters. We confirm that green innovation characterized by digital technology is an important path for enterprises to achieve clean production and green manufacturing upgrades by finding the clean production effect of green innovation under enterprise digitalization conditions through the test, extending the boundaries of existing green innovation research and improving the theoretical outreach of green innovation performance.

## 2. Literature Review and Hypothesis Development

### 2.1. Enterprise Digitization and Green Innovation

Green innovation is usually related to the survival and development of businesses in a market characterized by intense competition [37]. Green innovation, also known as the innovations of green technologies, products and processes, refers to the pioneering creativity relating to products and technologies with the aim of seizing green competitive advantages while reducing environmental costs in an all-round way [38]. However, Chinese companies face huge challenges in green innovation. First of all, green innovation should be guaranteed by sufficient financial resources [39], but its highlighted balance between environment and economic benefits brings a hard-understood complexity that aggravates information asymmetry, thus placing innovative activities in a tricky financial situation [40]. Secondly, green innovation, with more uncertainties, will engage companies in higher risks [41], while managers are more likely to pursue short-term benefits [42].

From an innovation resource perspective, emerging economies are often financially constrained [30]. Additionally, green innovation involves so many complexities and uncertainties on a long-term basis that investors will be more sensitive to information asymmetry. On the other hand, green innovation, typically considered a public good, will generate positive externalities in the form of knowledge spillovers. To protect the achievements of green innovation, businesses may reduce or even fail on the disclosure of relevant information, making too much asymmetric information to have access to financial support from outside [43]. As a result, the limited access to external financing will supercharge the propensity to allocate scarce financial resources to daily operations and short-term investments instead of green creative activities [43]. Therefore, due to the noticeable complexities and uncertainties arising from green innovation [44], the financing constraints caused by information asymmetry will become a major barrier [43].

Digital transformation is a solution that can help mitigate information asymmetry and increase access to the financial resources necessary for green innovation. According to signaling theory, conducting digital transformation gives a positive signal of better prospects and potential to outside investors; therefore, the company may become more attractive in a capital market. Meanwhile, by taking advantage of digital, transparent and intelligent information inspired by digital transformation in every process of production [45], managers can not only swiftly access high-quality information but also effectively deliver readily understandable information to targeted parties outside at a low cost, thus enhancing information transparency, mitigating information asymmetry both inside and outside, as well as reducing external transaction costs and the difficulty in the access to financing aids. With abundant financial resources, companies will be more likely to undertake social responsibilities [46] and will opt for sustainability-oriented green innovation activities.

Moreover, digital transformation will help businesses increasingly grow risk-tolerant, thus intensifying the allocation of resources to green innovation. Compared to other typical technological innovations, green innovation, identified by the nature of greater uncertainties on a long-term basis, requires greater risk-taking [41]. According to the principal-agent theory, to avoid the risks of financial loss and dismissal caused by an investment failure, managers are inclined to engage in risk-off activities [42] without considering green innovation. From the perspective of corporate governance, the effective use of digital technologies can introduce transparent and visible performances to management and operation, wind down the risks of governance owing to information asymmetry, reduce managers’ speculations and the inappropriate pursuits of personal interests, as well as help channel resources to green innovation. Viewed from the perspective of risks, risk-taking is a determinant of sustainable green innovation [47,48]. Digital technologies that effectively disseminate will improve information transparency and sharing [49] and help shareholders, creditors and other major interested parties to reach a consensus on risk identity with the executives when communications cast off the shackles subject to space and time, thus improving their risk tolerance [50,51,52].

Companies can improve the efficiency of resource allocation and coordination with the help of digital technology and gain access to a large amount of external information and knowledge, increasing the knowledge base of green innovation. Based on the information processing theory, digital technologies characterized by scalability and openness can help operators identify their requirements for resources, promote communication and exchange with other counterparts and find the valuable information concerned with green innovation effectively at a low cost. They can also enable businesses to process information more efficiently and build the ability to coordinate the resources upstream or downstream in a supply chain and the ability to quickly respond to volatile market demands [25]. Within the enterprise, by making full use of their strong capabilities of data mining and processing [13], the high level of digitalization enables enterprises to have easy access to their production and operation statuses and dynamic information related to the market in a timely, comprehensive and accurate manner. In a highly efficient digital system, managers are allowed to identify and assess the potential risks that arise during operation in advance and produce responsive strategies in time. Therefore, higher risk-taking will work to influence executives’ risk and investment preferences and dampen the exclusive pursuits of economic benefits, leading to sustainable green innovation.

In summary, digitalization can first mitigate information asymmetry inside and outside of a company, facilitating access to financial resources and thus reducing the financial constraints on green innovation. In addition, corporate digitization can optimize resource allocation and improve risk-taking, thus sustainably nurturing green innovation. Therefore, the following hypotheses are proposed:

**Hypothesis 1:**  *Digital transformation can effectively promote corporate green innovation.*

**Hypothesis 2:**  *Digital transformation can promote corporate green innovation by easing financing constraints and increasing risk-taking.*

### 2.2. Moderation Effects

#### 2.2.1. The Level of Economic Development

Due to the differences in the natural geographical environment and development basis, there is a great imbalance in the level of economic development among different regions in China, which may lead to various impacts of digitization on green innovation resources in different regions. On the one hand, people in economically developed regions have a strong desire for product quality and environmental awareness [34] and can also afford green innovation premiums. Digitization can provide necessary innovation resources for green innovation in time. On the other hand, in economically developed areas with rich innovation resources and base [35,36], digitization can improve the efficiency and technical capability of green innovation. Therefore, Digitization may strengthen the positive impact of economic development on green innovation. We propose the following hypothesis:

**Hypothesis 3a:**  *The impact of digitization on green innovation is stronger in regions with a higher level of economic development.*

#### 2.2.2. The Intensity of Environmental Regulation

According to the technical innovation theory and the environmental regulation theory, the intensity of environmental regulation is an important factor influencing the green innovations of local businesses [53]. Enterprises in regions with stronger environmental regulation face greater pressure and impact on environmental protection; the resulting high costs of environmental pollution may be crippling for businesses, which will force them to resort to more green creativities; that is, the businesses suffering from economic losses owing to environmental pollution will be compensated or even benefit from the “innovation compensation” mechanism [54]. Therefore, digitization may enhance the positive impact of the regulatory environment on green innovation. We propose the following hypothesis:

**Hypothesis 3b:**  *The impact of digitization on green innovation is stronger in regions with a high intensity of environmental regulation.*

#### 2.2.3. The Degree of Intellectual Property Protection

According to new institutional economics, intellectual property (IP) protection can influence corporate creativity by reducing the financial uncertainties of R&D and enhancing the effective allocation of R&D resources [55,56]. A company’s monopoly returns coming from innovation achievements are directly determined by the degree of IP protection [57]; that is, a region with a greater degree of intellectual property protection can constrain the replication and dissemination of innovation achievements by other innovation subjects more effectively, further protecting green creative achievements and raising expected returns for companies—a strong incentive for firms’ green innovative activities. Therefore, the positive impacts of digitalization on green innovation may vary with the different degrees of IP protection. We propose the following hypothesis:

**Hypothesis 3c:**  *The impact of digitization on green innovation is stronger in regions with a greater the degree of intellectual property protection.*

#### 2.2.4. The Nature of Ownership

There are significant differences between state-owned enterprises and non-state-owned enterprises in terms of resource access and relations with the government. By comparison, the state-owned enterprises in China are more closely tied to the government than the other sectors, playing dual roles as market players in the economy with a political identity [58]. In the period of transition to a green economy in China, state-owned enterprises seem to play a leading role in undertaking environmental responsibilities; on the other hand, their close connection with the government is advantageous for them due to receiving increasing policy supports and priority [59], creating better conditions for developing green innovations. Therefore, during digital transformation, state-owned enterprises are more motivated and can carry out innovative green activities more efficiently than their non-state-owned counterparts. Therefore, the following hypothesis is proposed:

**Hypothesis 3d:**  *The impact of digitization on green innovation is more significant in state-owned enterprises than non-state-owned enterprises.*

#### 2.2.5. The Characteristics of Industry

Due to various industries’ having varying contributions in terms of pollution, the effect of promoting digitalization on green innovation may be heterogeneous. Heavily polluting industries are often faced with serious doubts about whether they pay attention to environmental issues and fulfill their social responsibilities [60]. At the same time, a series of mandatory regulatory policies and documents issued for heavily polluting industries have placed them under greater scrutiny and pressure for ecological protection [61]. In order to relieve the pressure of environmental regulation and seek a sustainable development path, heavily polluting enterprises will actively seize on the opportunities of digital transformation to strengthen their green innovations. Therefore, the following hypothesis is proposed:

**Hypothesis 3e:**  *The impact of digitization on green innovation is stronger in heavily polluting industries.*

### 2.3. The Environmental Value

If digitization promotes green innovation, then it is necessary to judge the environmental value of an enterprise’s green behavior, especially for enterprises in heavily polluting industries. As the major emitters of pollutants, heavily polluting industries are under close supervision and have raised considerable concern for the government and the public. Faced with the great pressure and high costs arising from ecologically regulated activities, it is imperative for heavy polluters to seek the way for cleaner production. Green technology innovation, as the solution, can achieve corporate energy conservation and emission reductions [62,63,64]. Digitization can improve the efficiency of resource allocation and coordination and help enterprises to continuously apply the environmental protection strategy to the production process and products. The continued implementation of the strategy can not only effectively reduce pollutant emission and environmental costs but also may promote the maximization of social and economic benefits. In other words, green innovation may become one channel for digitization to promote cleaner production. Therefore, the following hypothesis is proposed:

**Hypothesis 4:**  *Green innovation characterized by digitization can facilitate cleaner production.*

## 3. Research Design

### 3.1. Sample and Data Sources

In this study, we take Chinese A-share listed companies from 2011 to 2019 as our primary sample. Green innovation in a company is evaluated by analyzing its green patents. We first obtained corporate patent data (applied and granted) from the China Research Data Service Center (CNRDS). Then, we identify the green patent by using the code of each patent to match the code from the green innovation list in the International Patent Classification Code (IPC). After this procedure, we identify the number of green patent applications and the number of granted green patents for each firm every year.

The corporate annual reports and governance data were obtained from the China Stock Market & Accounting Research (CSMAR) database. For enterprise digitization, according to the detailed items of intangible assets at the end of the year in the notes to the financial report’s mandatory disclosure by the listed company, we manually identified the details related to digitization such as software, Internet, the client, a management system, an intelligent platform, etc., and added them up to calculate the amount of the corresponding detailed items-amount of intangible assets related to digitization. In the robustness test, digitization is alternatively gauged based on the frequencies which are calculated by doing a textual analysis on the management discussion (MD & A) text of the company’s annual report through machine learning. Firstly, due to the lack of a specialized dictionary of terms in the field of digital economy, this paper constructs a dictionary of enterprise digital terms based on the semantic system of national policy. Manually screening 30 important national level digital economy documents (“Notice of The State Council on the issuance of an Action Plan to Promote the Development of Big Data”,” A guideline on promoting the development of “Internet +” smart energy”, etc.) from the website of the Central People’s Government, the Ministry of Industry and Information Technology to extract keywords related to enterprise digitization through Python and artificial identification, 238 words (Internet, AI, IT, etc.) related to digital transformation with a retention frequency of more than or equal to five times are retained, which constitute the digital term dictionary of this paper. Secondly, we add the 238 words in the above digital term dictionary to the “jieba” Chinese dictionary of the Python software package and then do a textual analysis on the management discussion (MD & A) part of the company’s annual report to measure of digitization at the firm-year level.

The regional economic data stemmed from the Statistical Yearbook of each province. Our sample was processed as follows: (1) the samples in financial sectors are removed; (2) the sampled companies marked with ST, ST * and PT are counted out; and (3) the sampled companies with incomplete financial data are excluded. By doing so, our final sample includes 13,140 firm-year observations from across 2636 listed companies. The details of sample selection procedures are listed in Table 1. Additionally, all of the continuous variables were winsorized at the 1% and 99% levels.

### 3.2. Variables

#### 3.2.1. Dependent Variable

In this paper, we follow the method from previous studies [65,66] and use green patents to measure green innovation activities. The reason is that green patents are the outcomes of green innovation activities. We use the numbers of the applications for and the authorizations of green patents to evaluate the quantity and quality of corporates’ green innovation, respectively. In the case of no green patents in a sampled company, green innovation was then measured by using the natural logarithm of one plus the number of applications for green patents and with the natural logarithm of one plus the number of the granting of green patents, which are expressed by *lnAGreen* and *lnGGreen*, respectively.

#### 3.2.2. Independent Variable

The independent variable in this study is enterprise digitalization. A firm’s digitization is a systematic process calling for a wide range of applications of digital technologies, a great deal of investment and the construction of modern IT systems, forming data, software and technologies. It contributes to the part of intangible assets in financial statements, and the amount is in the details of the intangible assets. In our main regression, we manually identify the details of intangible assets and calculate the sum of digitalized intangible assets. We measure the degree of green innovation as the proportion of the digitally related intangible assets to the total intangible assets.

#### 3.2.3. Mediator Variables

There are two mediator variables in this paper: financial constraints and risk-taking. First, we follow the methodology from Hadlock and Pierce and use the SA index to measure the financial constraints [67]. The larger SA index represents the stronger financial constraints. Second, we follow the methodology of Koerniadi et al. [68] and use the following specification to measure risk-taking (*CRT*):

(1) $$CRT_{i,y}=ln\left[\sqrt{\frac{1}{T}\overset{T}{\underset{t=1}{\sum }}{\left(r_{i,y,t}-\frac{1}{T}\overset{T}{\underset{t=1}{\sum }}r_{i,y,t}\right)}^{2}}\right]$$

where *r_i,y,t_* is the rate of returns from the stock of the company *i* after its reinvestment with cash dividends on the day *t* in the year *y*, and *T* means the number of trading days for which the company has returns from stock in year *y*.

#### 3.2.4. Moderator Variables

The moderator variables in this study include the level of economic development (*GDP*), the intensity of environmental regulation (*ER*), the degree of intellectual property protection (*IP*), the nature of ownership (*SOE*) and the characteristics of the industry (*Pollute*). The detailed definitions of these variables are reported in Table 2.

#### 3.2.5. Cleaner Production

We use green total factor productivity to measure the cleaner production effects of green innovations characterized by the digitization. The reason is that green total factor productivity is established to maximize social and economic benefits by introducing energy consumption and emissions. Referring to the methodology of Tone [69], *GTFP* is evaluated with the global Malmquist–Luenberger (GML) index based on the slacks-based measure (SBM) of directional distance function—an evaluation that can considerably improve the accuracy. The detailed calculation steps as well as the specification are shown in Tone [69].

#### 3.2.6. Control Variables

In this paper, we controlled the firm-level variables in terms of finance and governance that may affect green innovation [5,70]. Specifically, the control variables interpreting finance include company size (*Size*), the return on assets (*Roa*), liabilities–assets ratio (*Lev*), corporate growth (*Growth*), business value (*TobinQ*) and the cash flow from operation (*Cfo*). Additionally, the control variables interpreting governance include CEO duality (*Dual*), Ownership Concentration (*Top1*), board size (*Board*), the proportion of independent directors (*Indep*) and market concentration (*HHI*). At the same time, in considering that green innovation is also affected by the macroeconomic environment, the regional economic indicator—regional economic development (*lnGDP*)—is also under control in this model. Please see Table 2 for more details relating to the definitions.

### 3.3. Empirical Model

To examine the impacts of digital transformation on green innovation, our empirical model was developed as follows:

(2) $$Green_{i,t}=\alpha_{0}+\alpha_{1}Digital_{i,t}+\alpha_{i}Controls+\sum YEAR+\sum INDUSTRY+\varepsilon_{i,t}$$

where the dependent variable of *Green* represents corporate green innovation, the independent variable of *Digital* indicates corporate digitalization and *Controls* is the set of control variables. *Year* and *Industry* are the year and industry fixed effects, respectively, and ε is the random error term.

To further describe and test the transmission path from digitalization to green innovation, we used stepwise regression [71] to construct the following models:

(3) $$M_{i,t}=\alpha_{0}+\alpha_{1}Digital_{i,t}+\alpha_{i}Controls+\sum YEAR+\sum INDUSTRY+\varepsilon_{i,t}$$

(4) $$Green_{i,t}=\alpha_{0}+\alpha_{1}Digital_{i,t}+\alpha_{2}M_{i,t}+\alpha_{i}Controls+\sum YEAR+\sum INDUSTRY+\varepsilon_{i,t}$$

where *M* is the mediation for the path from digitalization to green innovation, expressed by financial constraints (*SA*) and risk-taking (*CRT*), respectively, and the rest of the variables are identified as the same present in Model (2).

We also constructed the following Model (5) to test the cleaner production effect of green innovation characterized by the digitization:

(5) $$\begin{array}{c} GTFP_{it}=\delta_{0}+\delta_{1}Digital_{it}\times Green_{it}+\delta_{2}Digital_{it}+\delta_{3}Green_{it}+\delta_{4}Control\\ +\sum YEAR+\sum INDUSTRY+\varepsilon_{i,t}\; \end{array}$$

where *GTFP* is green total factor productivity established by introducing energy consumption and emission, *Digital* × *Green* is the interaction between digitalization and green innovation and $\delta_{1}$ is the concerned coefficient. The other variables are the same as defined in Model (2).

## 4. Empirical Results

### 4.1. Descriptive Statistics

The descriptive statistics of the major variables are reported in Table 3, in which Panel A shows the descriptive statistics of the whole sample: the means of the applications for (*lnAGreen*) and the authorizations of (*lnGGreen*) green patents are 0.457 and 0.343, respectively, both with zero for the median value, implying that low-level green innovations are seen among the sampled companies; the mean of digitalization (*Digital*) is 0.113 with a standard deviation of 0.236; the maximum and minimum values are 0 and 1, respectively, showing that the average proportion of digitally related intangible assets is 11.3% in the sampled companies, which suggests that digitalization varies greatly among different companies and that there is much leeway for Chinese companies in an effort to conduct digital transformation.

Panel B shows a univariate analysis in which the samples are divided into two groups according to the median of digitalization. The numbers of green patent applications and authorizations of the highly digitalized companies are significantly greater than that of the low-digitalized group at the 1% level. Therefore, Hypothesis 1 is preliminarily verified.

### 4.2. Baseline Results

Based on Model (2), the results of the regression interpreting the relationship between corporate digitalization and innovation are seen in Table 4. The empirical results show that the regression coefficients interpreting the digitalization–application relations (*lnAGreen*) and the digitalization–authorization relations (*lnGGreen*) are significantly positive at the 5% level at least. With other influencing factors under control, a standard deviation increase in *Digital* is associated with the increase in *lnAGreen* and *lnGGreen* by 5.47% and 3.78%, respectively, indicating that digitalization can significantly promote green patent applications and authorizations. Furthermore, compared to the applications for green patents, hard-won authorizations often involve greater technical difficulties that need to be surmounted. Generally speaking, with higher digitalization, both the quantity and quality of green innovation will be significantly improved. Hypothesis 1 is supported.

### 4.3. Mediation Tests

The empirical results with corporate financial constraints (*SA*) as the mediation are shown in Table 5. In Column (1), the regression coefficient for *Digital* is significantly negative at the 5% level, implying that a company’s digitization can alleviate its financial constraints. In Columns (2) and (3), the regression coefficients for *SA* are significantly negative. It indicates that that financial constraints work as a partial mediation in the path from digitalization to green innovation, consistent with our expectations.

The empirical results with risk-taking (*CRT*) as the mediation are shown in Table 6. In Column (1), the regression coefficient for digital transformation is significantly positive at the 1% level, suggesting that digital transformation can help a company increasingly grow risk-tolerant. In Columns (2) and (3), the regression coefficients for risk-taking (*CRT*) are significantly positive, indicating that corporate risk-taking works as a partial mediation in the path from corporate social responsibility to green innovation. Generally, a company’s green innovation can, through a higher degree of risk that it is willing to endure, be intensified by its digitalization.

### 4.4. Moderation Analyses

#### 4.4.1. The Level of Economic Development

In this study, the samples are divided into a high economic development group and a low economic development group based on the annual industry median value of the level of economic development before the regressions are performed. The regression results in Table 7 show that the coefficient for *Digital* in the high economic development group is significantly larger than that in the low economic development group. It suggests that the economic development can strengthen the impact of digitalization on green innovation, consistent with our expectation. Therefore, H3a is validated.

#### 4.4.2. The Intensity of Environmental Regulation

The moderation effect of environmental regulation on our baseline findings are reported in Table 8. We find that the coefficient for *Digital* is significantly positive in the intensely regulated group, while the coefficient is not significant in the moderately regulated group, suggesting that the more intense the environmental regulation, the greater the positive effects of digitalization on green innovation, which is consistent with Hypothesis 3b.

#### 4.4.3. The Degree of Intellectual Property Protection

The results in Table 9 show the moderation effect of intellectual property protection. We find that the coefficients for *Digital* are significantly positive for high IP protection, while such coefficients are relatively lower and less significant in another group—among which the coefficient interpreting the relations between green patent applications (*lnAGreen*) and *Digital* is significantly positive, and the coefficient interpreting the relations between green patent authorizations (*lnGGreen*) and *Digital* is positive but not significant. This shows that the more intensive the use of intellectual property protection, the greater the positive effects of digitalization on green innovation, which is consistent with Hypothesis 3c.

#### 4.4.4. The Nature of Ownership

In this study, the samples were divided into state-owned and non-state-owned companies. The regression results in Table 10 show that the coefficient for *Digital* is significantly positive in the state-owned group, while for the non-state-owned group, it is not significant, indicating that digitization effectively improved the quantity and quality of green innovation in state-owned enterprises, which is consistent with Hypothesis 3d.

#### 4.4.5. The Characteristics of Industry

To test the moderation effects of various industries that have varying contributions to pollution on the relationship between digitization and green innovation, the samples were classified into heavily polluting industries and other industries. The regression results in Table 11 show that the coefficients for *Digital* are significantly positive in relation to the heavy polluters, while such coefficients are relatively lower and less significant in another group, among which the coefficient interpreting the relations between green patent applications (*lnAGreen*) and *Digital* is significantly positive, and the coefficient interpreting the relations between green patent authorizations (*lnGGreen*) and *Digital* is positive but not significant. This shows that the positive effects of digital transformation on green innovation are more pronounced among heavy polluters, which is consistent with Hypothesis 3e.

### 4.5. The Environmental Value

The regression results of Model (5) in Table 12 show that the coefficient of the interaction between digital transformation and green patent applications (*Digitalit* × *lnAGreenit*) is positive but not significant. It indicates that green patent application may become a channel for digitalization to promote cleaner production. However, this effect is not obvious because patent applications without novelty, creativity and practicability cannot be authorized by intellectual property authorities. The coefficient for the interaction between digital transformation and green patent authorizations (*Digitalit* × *lnGGreenit*) is significantly positive. It indicates that digitization can significantly promote cleaner production of enterprises through green innovation with significant technological advantages. The above results are consistent with Hypothesis 4.

## 5. Robustness Tests

### 5.1. Heckman Two-Stage Regression

In this study, the Heckman two-stage approach [72] was employed to address the self-selection-bias-induced endogenous problems arising from the fact that digital transformation may be decided concurrently with green innovation. In the first stage, a dummy variable for digitalization (*Digital_Dummy*), which is measured by the digitalization median of the sampled companies in the year, is involved in the Probit regression as the explanatory variable. At the same time, referring to Xu et al. [73], the exclusion restriction in the instrumental variable of *MeanDigital*—the mean value of the digitalization variable of other companies in the same industry in the same year—is imposed in the model, and the inverse Mills’ ratio (*IMR*) is derived. In the second stage, based on Model (1), the *IMR* is included in the regression.

The results of the two-stage regression can be seen in Table 13, where in Column (1), the regression coefficient for *MeanDigital* is significantly positive at the 1% level, which suggests that the digitization of other companies in the same industry in the same year has a positive impact on that of the sampled companies. In Column (2) and Column (3), the regression results with *IMR* show that the regression coefficients for digital transformation remain significantly positive, which is consistent with the foregoing conclusion even after the selection bias is under control.

### 5.2. Entropy Balancing Matching

Considering that the initial conditions of enterprises with different degrees of digital transformation are different, we follow Hainmueller [74] and use the entropy balance-matching method to eliminate the endogeneity problems that may be caused by sample self-selection. First, the samples are divided into treatment and control groups according to the annual industry median value of the digitalization variable. Second, this paper selects all the control variables as covariables for entropy balance matching. Finally, we take regression analysis based on the weights that result from entropy balancing matching. The covariate adjustment results are shown in Table 14. It indicates that after weighing using the entropy balance method, the mean, variance and skewness of the covariates of the treatment group and the control group are all close to the same. The regression results are shown in Column (1) and Column (2) of Table 15, suggesting that our main findings are robust.

### 5.3. Lagged Independent Variable

There is a possible reverse causality between digitalization and green innovation in the model. On the one hand, firms that have higher digital levels can promote green innovation. On the other hand, green innovation may also weigh on the momentum of high-level digitalization, sustainably fueling green transformation and upgrading in turn. The results of the regression, based on Model (2) with the independent variable *Digital* at one lag in Column (3) and Column (4) of Table 15, show that the coefficient for the lagged *Digital*_−1_ is significantly positive. The conclusions remain valid.

### 5.4. Alternative Measure of Independent Variable

To examine the robustness of our baseline findings, a company’s digital level is, alternatively, gauged by the total frequencies of all the digitally related words appearing in the MD & A text of the company’s annual report—expressed by the natural logarithm of one plus the total frequencies (*Digital1*) and the proportion of the total frequencies to the total paragraph length of the MD & A text (*Digital2*), respectively. The results are shown in Table 16, in which the regression coefficients for *Digital1* and *Digital2* are significantly positive at the 1% level, indicating that corporate digitalization has a vital role in promoting green innovation. The results of this study are proved again.

### 5.5. Alternative Empirical Specification

Since the outcomes of the green patent applications or the authorizations of the sampled companies characterized by lots of zeros may lead to regression bias, the Tobit model is applied in this study to re-estimate the impacts of digital transformation on green innovation. The results based on Model (2) are shown in Columns (1) and (2) of Table 17 with reference to the study by Moser and Voena [75] on the control for the year × industry fixed effects—a control for the changes in industries over time; that is, the influences of various industries with differences in time trends on green innovation are under control. The regression results are shown in Columns (3) and (4) of Table 17. According to the results of Table 17, our baseline findings remain robust.

## 6. Conclusions

As a crucial strategy for a business’s sustainable growth, green innovation may be stifled by information asymmetry and resource constraints. From the perspective of resource allocation to green creativity, we theoretically reveal the relationship between digitalization and green innovation in emerging economies. We find that corporate digitization has a significantly positive impact on green innovation and that the ease of financial constraints and higher risk-taking work as the mediations in the digitization–green innovation path. In addition, we find that the positive relationship between enterprise digitization and green innovation is stronger in regions with stronger economic development, stronger environmental regulation, higher intellectual property protection, as well as in state-owned and heavily polluting enterprises. This study introduces novelties to the present literature related to the digitization–green innovation relationship, complementary to the digitization–green innovation mechanism, and helping businesses further develop necessary incentives for green creative activities.

There are several limitations in our study. First, regarding the measurement of the enterprise digitalization, there is no unified standard and requirement for enterprise digitization disclosure. Although we follow the literature and use two widely used methods to measure enterprise digitization, both measures rely on subjective judgment, which may bias our results. We will continue to pay attention to the latest research progress of enterprise digitalization and continuously improve the indicator to provide more robust conclusions for subsequent studies. Second, different firms alternate and replace old and new technologies at different times. Thus, the heterogeneity of different corporate characteristics and their life cycle can be further explored in the relation between digitalization and green innovation. Third, this study only examines the relationship between digitalization and green innovation in China, while future studies can investigate this relationship in other emerging markets or using multinational data and explore possible changes and causes of this relationship in different institutional environments.

## Figures and Tables

**Table 1:** Sample selection process.

Number of A-share listed companies in Shanghai and Shenzhen from 2011–2019 (firm-years)	27,424
-Observations are within the financial industry	(784)
-Observations belong to ST, ST * and PT companies	(2202)
-Observations missing data on key variables	(11,298)
Final sample size	13,140

Note: This table reports the details of sample selection procedures.

**Table 2:** Definitions of variables.

Variable Type	Symbols	Definitions
Dependent variables	*lnAGreen*	Ln (number of green applied patents + 1)
	*lnGGreen*	Ln (number of green granted patents + 1)
Independent variable	*Digital*	Intangible assets related to digital transformation/Total intangible assets
Mediator Variables	*SA*	SA index, detail computing formula referring the methodology of Koerniadi et al. (2010) [68]
	*CRT*	Detail computing formula. See Model (1)
Moderator Variables	*GDP*	Gross domestic product in regions
	*ER*	Pollution control costs/Added value of the secondary industry
	*IP*	Ln [(the number of intellectual property infringement cases/the total population + the number of regional lawyers/the total population)/2]
	*SOE*	A dummy variable that equals 1 if a firm is a state-owned enterprise and 0 otherwise
	*Pollute*	A dummy variable that equals 1 if an industry is heavily polluted and 0 otherwise
Cleaner production	*GTFP*	Measured by green total factor productivity (GTFP). Calculation details referring to the methodology of Tone (2001) [69]
Control variables	*Size*	The natural logarithm of total assets
	*Roa*	Net profit/Total assets
	*Lev*	Total liabilities/Total assets
	*Growth*	(Operating income in year *t* + 1 − Operating income in year *t*)/Operating income in year *t*
	*Cfo*	Operating cash flow/Total assets
	*TobinQ*	Market value/Capital replacement cost
	*Dual*	A dummy variable with a value of 1 if the CEO and chairman is the same person and 0 otherwise
	*Top1*	Shareholding ratio of the first shareholder
	*Board*	The natural logarithm of total number of board of directors
	*Indep*	Number of independent directors/Total number of board of directors
	*HHI*	HHI index of the industry
	*lnGDP*	The natural logarithm of GDP of the province of the company’s location

**Table 3:** Descriptive statistics.

Panel A: Descriptive Statistics of the Full Sample						
Variables	Obs	Mean	SD	Median	Min	Max
*lnAGreen*	13,140	0.457	0.860	0	0	3.807
*lnGGreen*	13,140	0.343	0.709	0	0	3.296
*Digital*	13,140	0.113	0.236	0.023	0	1
*SA*	13,140	3.522	0.301	3.475	2.966	4.158
*CRT*	13,140	−3.609	0.308	−3.624	−4.372	−2.847
*GDP*	13,140	43,779.730	27,442.010	35,478.090	605.830	107,671.100
*ER*	11,928	0.002	0.002	0.002	0.000	0.025
*IP*	13,140	0.471	0.705	0.332	−1.189	2.081
*SOE*	13,140	0.370	0.483	0	0	1
*Pollute*	13,140	0.202	0.401	0	0	1
*Size*	13,140	22.220	1.259	22.060	20.020	26.150
*Roa*	13,140	0.040	0.055	0.0379	−0.224	0.194
*Lev*	13,140	0.426	0.206	0.417	0.050	0.893
*Growth*	13,140	0.195	0.451	0.114	−0.509	3.073
*Cfo*	13,140	0.047	0.067	0.046	−0.156	0.234
*TobinQ*	13,140	2.140	1.310	1.708	0.907	8.362
*Dual*	13,140	0.263	0.440	0	0	1
*Top1*	13,140	35.260	14.710	33.780	9	72.880
*Board*	13,140	2.133	0.201	2.197	1.609	2.708
*Indep*	13,140	0.375	0.053	0.353	0.333	0.571
*HHI*	13,140	0.147	0.165	0.090	0.024	1
*lnGDP*	13,140	10.460	0.717	10.480	8.064	11.590
**Panel B: Partitioned by the Degree of Digital Transformation**						
**Variables**	**(1) High Degree of Digitalization**		**(2) Low Degree of Digitalization**			**(1)–(2)**
	**Obs**	**Mean**	**Obs**		**Mean**	**Mean-diff**
*lnAGreen*	6570	0.522	6570		0.392	0.130 ***
*lnGGreen*	6570	0.381	6570		0.305	0.076 ***

Note: *** represent significance at the 1% level, respectively, and the *t*-values are in brackets.

**Table 4:** Baseline Regression Results.

Variable	(1)	(2)
	*lnAGreen*	*lnGGreen*
*Digital*	0.106 ***	0.055 **
	(3.199)	(1.997)
*Size*	0.167 ***	0.134 ***
	(21.289)	(20.618)
*Roa*	0.935 ***	0.363 ***
	(6.148)	(2.883)
*Lev*	0.191 ***	0.111 ***
	(4.085)	(2.878)
*Growth*	−0.056 ***	−0.040 ***
	(−3.535)	(−3.113)
*Cfo*	−0.038	0.070
	(−0.331)	(0.746)
*TobinQ*	−0.002	−0.006
	(−0.385)	(−1.092)
*Dual*	0.058 ***	0.039 ***
	(3.613)	(2.937)
*Top1*	−0.001 *	−0.000
	(−1.953)	(−1.215)
*Board*	0.076 *	0.094 ***
	(1.729)	(2.578)
*Indep*	0.040	0.285 **
	(0.254)	(2.190)
*HHI*	−0.552 ***	−0.383 ***
	(−10.503)	(−8.827)
*lnGDP*	0.110 ***	0.085 ***
	(10.403)	(9.747)
Constant	−4.665 ***	−3.891 ***
	(−20.367)	(−20.548)
Year fixed effects	Yes	Yes
Industry fixed effects	Yes	Yes
Observations	13,140	13,140
Adj. R-squared	0.174	0.170

Note: *, ** and *** represent significance at the 10%, 5% and 1% levels, respectively, and the *t*-values are in brackets.

**Table 5:** Mediation tests: financial constraints.

Variable	(1)	(2)	(3)
	*SA*	*lnAGreen*	*lnGGreen*
*SA*		−0.325 ***	−0.268 ***
		(−12.470)	(−12.459)
*Digital*	−0.022 **	0.099 ***	0.049 *
	(−2.016)	(2.998)	(1.789)
*Size*	0.035 ***	0.178 ***	0.143 ***
	(13.327)	(22.713)	(22.041)
*Roa*	−0.196 ***	0.871 ***	0.310 **
	(−3.866)	(5.759)	(2.478)
*Lev*	0.246 ***	0.271 ***	0.177 ***
	(15.826)	(5.778)	(4.574)
*Growth*	−0.016 ***	−0.061 ***	−0.045 ***
	(−2.984)	(−3.880)	(−3.454)
*Cfo*	0.060	−0.018	0.087
	(1.578)	(−0.161)	(0.922)
*TobinQ*	0.015 ***	0.002	−0.002
	(6.891)	(0.362)	(−0.348)
*Dual*	−0.105 ***	0.024	0.011
	(−19.560)	(1.482)	(0.814)
*Top1*	−0.002 ***	−0.002 ***	−0.001 ***
	(−14.868)	(−3.554)	(−2.817)
*Board*	−0.005	0.074 *	0.092 **
	(−0.312)	(1.705)	(2.559)
*Indep*	−0.206 ***	−0.027	0.230 *
	(−3.926)	(−0.172)	(1.774)
*HHI*	0.044 **	−0.537 ***	−0.372 ***
	(2.526)	(−10.287)	(−8.602)
*lnGDP*	−0.062 ***	0.090 ***	0.068 ***
	(−17.721)	(8.433)	(7.783)
Constant	3.404 ***	−3.559 ***	−2.978 ***
	(44.569)	(−14.565)	(−14.739)
Year fixed effects s	Yes	Yes	Yes
Industry fixed effect	Yes	Yes	Yes
Observations	13,140	13,140	13,140
Adj. R-squared	0.249	0.184	0.179

Note: *, ** and *** represent significance at the 10%, 5% and 1% levels, respectively, and the *t*-values are in brackets.

**Table 6:** Mediation tests: risk-taking.

Variables	(1)	(2)	(3)
	*CRT*	*lnAGreen*	*lnGGreen*
*CRT*		0.077 **	0.072 ***
		(2.329)	(2.656)
*Digital*	0.029 ***	0.104 ***	0.053 *
	(3.250)	(3.132)	(1.921)
*Size*	−0.075 ***	0.173 ***	0.139 ***
	(−35.985)	(21.011)	(20.470)
*Roa*	−0.143 ***	0.946 ***	0.373 ***
	(−3.533)	(6.218)	(2.964)
*Lev*	0.168 ***	0.178 ***	0.099 **
	(13.521)	(3.784)	(2.547)
*Growth*	0.046 ***	−0.059 ***	−0.044 ***
	(11.096)	(−3.744)	(−3.355)
*Cfo*	−0.096 ***	−0.030	0.077
	(−3.154)	(−0.266)	(0.819)
*TobinQ*	0.013 ***	−0.003	−0.007
	(7.685)	(−0.541)	(−1.268)
*Dual*	0.018 ***	0.057 ***	0.038 ***
	(4.222)	(3.526)	(2.838)
*Top1*	0.000 **	−0.001 **	−0.001
	(2.210)	(−1.998)	(−1.267)
*Board*	−0.048 ***	0.080 *	0.097 ***
	(−4.123)	(1.812)	(2.672)
*Indep*	−0.018	0.041	0.286 **
	(−0.421)	(0.262)	(2.200)
*HHI*	0.028 **	−0.554 ***	−0.385 ***
	(2.014)	(−10.544)	(−8.875)
*lnGDP*	0.003	0.110 ***	0.085 ***
	(1.069)	(10.382)	(9.724)
Constant	−2.116 ***	−4.503 ***	−3.739 ***
	(−34.740)	(−18.816)	(−18.895)
Year fixed effects	Yes	Yes	Yes
Industry fixed effect	Yes	Yes	Yes
Observations	13,140	13,140	13,140
Adj. R-squared	0.545	0.174	0.170

Note: *, ** and *** represent significance at the 10%, 5% and 1% levels, respectively, and the *t*-values are in brackets.

**Table 7:** Moderation tests: level of economic development.

Variables	High	Low	High	Low
	(1)	(2)	(3)	(4)
	*lnAGreen*	*lnAGreen*	*lnGGreen*	*lnGGreen*
*Digital*	0.129 ***	0.091 *	0.081 **	0.031
	(2.812)	(1.854)	(2.156)	(0.760)
*Size*	0.177 ***	0.162 ***	0.142 ***	0.129 ***
	(15.766)	(14.444)	(15.450)	(13.850)
*Roa*	0.855 ***	1.094 ***	0.246	0.540 ***
	(4.099)	(4.887)	(1.439)	(2.887)
*Lev*	0.235 ***	0.115 *	0.130 **	0.074
	(3.573)	(1.709)	(2.417)	(1.326)
*Growth*	−0.068 ***	−0.049 **	−0.037 **	−0.048 ***
	(−3.015)	(−2.225)	(−2.019)	(−2.617)
*Cfo*	0.102	−0.200	0.265 **	−0.147
	(0.644)	(−1.207)	(2.037)	(−1.064)
*TobinQ*	0.010	−0.015 *	0.008	−0.021 ***
	(1.034)	(−1.691)	(1.006)	(−2.699)
*Dual*	0.077 ***	0.044 *	0.049 ***	0.033
	(3.545)	(1.794)	(2.762)	(1.602)
*Top1*	−0.001 *	−0.001	−0.000	−0.001
	(−1.826)	(−1.162)	(−0.529)	(−1.312)
*Board*	0.115 *	0.010	0.145 ***	0.014
	(1.836)	(0.164)	(2.819)	(0.273)
*Indep*	−0.147	0.243	0.145	0.445 **
	(−0.646)	(1.107)	(0.777)	(2.426)
*HHI*	−0.572 ***	−0.525 ***	−0.378 ***	−0.384 ***
	(−7.739)	(−6.928)	(−6.235)	(−6.073)
Constant	−3.732 ***	−3.535 ***	−3.251 ***	−2.945 ***
	(−12.816)	(−12.777)	(−13.612)	(−12.745)
Year fixed effects	Yes	Yes	Yes	Yes
Industry fixed effects	Yes	Yes	Yes	Yes
Observations	7111	6029	7111	6029
Adj. R-squared	0.173	0.168	0.173	0.160
Empirical *p*-values	0.087 *		0.013 **	
F-test	21.34 ***		19.18 ***	

Note: *, ** and *** represent significance at the 10%, 5% and 1% levels, respectively, and the *t*-values are in brackets; Empirical *p*-values are used to test the significance of coefficient differences, which are obtained using Bootstrap with 1000 repeated samples; F test is used to examine the coefficient difference between groups as well.

**Table 8:** Moderation tests: environmental regulation.

Variables	Strong	Weak	Strong	Weak
	(1)	(2)	(3)	(4)
	*lnAGreen*	*lnAGreen*	*lnGGreen*	*lnGGreen*
*Digital*	0.123 ***	0.086	0.067 *	0.048
	(2.587)	(1.608)	(1.721)	(1.080)
*Size*	0.179 ***	0.144 ***	0.140 ***	0.131 ***
	(16.712)	(11.209)	(15.893)	(12.134)
*Roa*	0.970 ***	0.658 ***	0.543 ***	−0.011
	(4.654)	(2.779)	(3.174)	(−0.058)
*Lev*	0.243 ***	0.162 **	0.153 ***	0.066
	(3.851)	(2.217)	(2.950)	(1.077)
*Growth*	−0.082 ***	−0.047 **	−0.060 ***	−0.032 *
	(−4.005)	(−2.042)	(−3.574)	(−1.658)
*Cfo*	−0.028	0.067	0.113	0.165
	(−0.183)	(0.370)	(0.911)	(1.085)
*TobinQ*	0.012	−0.018 *	0.004	−0.015 *
	(1.328)	(−1.762)	(0.554)	(−1.759)
*Dual*	0.036 *	0.079 ***	0.010	0.071 ***
	(1.657)	(3.129)	(0.564)	(3.355)
*Top1*	−0.000	−0.001 *	0.000	−0.001
	(−0.123)	(−1.762)	(0.337)	(−0.819)
*Board*	0.036	0.140 **	0.053	0.132 **
	(0.596)	(2.038)	(1.082)	(2.305)
*Indep*	−0.074	0.379	0.255	0.416 **
	(−0.340)	(1.574)	(1.426)	(2.068)
*HHI*	−0.662 ***	−0.356 ***	−0.473 ***	−0.222 ***
	(−9.071)	(−4.262)	(−7.911)	(−3.175)
*lnGDP*	0.109 ***	0.119 ***	0.082 ***	0.093 ***
	(7.775)	(6.945)	(7.059)	(6.505)
Constant	−4.850 ***	−4.507 ***	−3.924 ***	−4.040 ***
	(−15.414)	(−12.271)	(−15.198)	(−13.156)
Year fixed effects	Yes	Yes	Yes	Yes
Industry fixed effects	Yes	Yes	Yes	Yes
Observations	6711	5217	6711	5217
Adj. R-squared	0.176	0.173	0.166	0.178
Empirical *p*-values	0.000 ***		0.000 ***	
F-test	20.30 ***		19.19 ***	

Note: *, ** and *** represent significance at the 10%, 5% and 1% levels, respectively, and the *t*-values are in brackets. Empirical *p*-values are used to test the significance of coefficient differences, which are obtained using Bootstrap with 1000 repeated samples; F test is used to examine the coefficient difference between groups as well.

**Table 9:** Moderation tests: intellectual property protection.

Variables	High	Low	High	Low
	(1)	(2)	(3)	(4)
	*lnAGreen*	*lnAGreen*	*lnGGreen*	*lnGGreen*
*Digital*	0.138 ***	0.088 *	0.073 **	0.048
	(3.147)	(1.731)	(1.989)	(1.145)
*Size*	0.175 ***	0.158 ***	0.137 ***	0.131 ***
	(16.659)	(13.136)	(15.743)	(13.320)
*Roa*	0.716 ***	1.204 ***	0.174	0.594 ***
	(3.416)	(5.453)	(0.998)	(3.279)
*Lev*	0.111 *	0.275 ***	0.018	0.199 ***
	(1.733)	(3.977)	(0.343)	(3.511)
*Growth*	−0.051 **	−0.064 ***	−0.038 *	−0.048 ***
	(−2.183)	(−3.091)	(−1.960)	(−2.783)
*Cfo*	0.107	−0.240	0.208	−0.101
	(0.683)	(−1.441)	(1.599)	(−0.742)
*TobinQ*	−0.003	−0.006	−0.009	−0.005
	(−0.376)	(−0.623)	(−1.184)	(−0.681)
*Dual*	0.064 ***	0.050 **	0.044 **	0.031
	(2.997)	(2.021)	(2.459)	(1.545)
*Top1*	−0.001 *	−0.001	−0.000	−0.001
	(−1.836)	(−1.186)	(−0.880)	(−0.997)
*Board*	0.025	0.163 ***	0.124 **	0.090 *
	(0.402)	(2.637)	(2.393)	(1.783)
*Indep*	−0.306	0.293	0.118	0.410 **
	(−1.390)	(1.302)	(0.648)	(2.214)
*HHI*	−0.527 ***	−0.535 ***	−0.396 ***	−0.336 ***
	(−7.179)	(−7.065)	(−6.496)	(−5.401)
*lnGDP*	0.086 ***	0.124 ***	0.061 ***	0.091 ***
	(4.947)	(8.271)	(4.267)	(7.402)
Constant	−4.400 ***	−4.808 ***	−3.738 ***	−3.914 ***
	(−13.287)	(−14.447)	(−13.596)	(−14.328)
Year fixed effects	Yes	Yes	Yes	Yes
Industry fixed effects	Yes	Yes	Yes	Yes
Observations	7354	5786	7354	5786
Adj. R-squared	0.193	0.161	0.193	0.152
Empirical *p*-values	0.005 **		0.006 ***	
F-test	23.54 ***		21.79 ***	

Note: *, ** and *** represent significance at the 10%, 5% and 1% levels, respectively, and the *t*-values are in brackets; Empirical *p*-values are used to test the significance of coefficient differences, which are obtained using Bootstrap with 1000 repeated samples; F test is used to examine the coefficient difference between groups as well.

**Table 10:** Moderation tests: the nature of ownership.

Variables	SOE	Non-SOE	SOE	Non-SOE
	(1)	(2)	(3)	(4)
	*lnAGreen*	*lnAGreen*	*lnGGreen*	*lnGGreen*
*Digital*	0.187 ***	0.046	0.125 ***	0.008
	(3.335)	(1.098)	(2.710)	(0.239)
*Size*	0.190 ***	0.153 ***	0.155 ***	0.123 ***
	(15.356)	(14.208)	(15.210)	(13.751)
*Roa*	0.233	1.212 ***	−0.048	0.514 ***
	(0.808)	(6.762)	(−0.201)	(3.450)
*Lev*	−0.095	0.377 ***	−0.100	0.275 ***
	(−1.238)	(6.215)	(−1.602)	(5.463)
*Growth*	−0.050 *	−0.060 ***	−0.046 **	−0.040 **
	(−1.931)	(−3.051)	(−2.173)	(−2.435)
*Cfo*	0.379 **	−0.183	0.353 **	−0.037
	(2.013)	(−1.271)	(2.286)	(−0.312)
*TobinQ*	0.016	−0.008	0.009	−0.009
	(1.269)	(−1.046)	(0.866)	(−1.451)
*Dual*	0.019	0.074 ***	0.005	0.043 ***
	(0.484)	(4.033)	(0.149)	(2.834)
*Top1*	−0.002 **	−0.001	−0.001	−0.000
	(−2.059)	(−1.213)	(−1.327)	(−0.480)
*Board*	0.138 **	−0.008	0.148 ***	0.046
	(2.108)	(−0.134)	(2.741)	(0.907)
*Indep*	0.383	−0.275	0.558 ***	0.067
	(1.586)	(−1.293)	(2.812)	(0.377)
*HHI*	−0.527 ***	−0.567 ***	−0.309 ***	−0.449 ***
	(−6.759)	(−7.513)	(−4.827)	(−7.159)
*lnGDP*	0.124 ***	0.095 ***	0.092 ***	0.068 ***
	(6.960)	(7.074)	(6.285)	(6.076)
Constant	−5.475 ***	−3.988 ***	−4.619 ***	−3.330 ***
	(−16.095)	(−12.218)	(−16.534)	(−12.274)
Year fixed effects	Yes	Yes	Yes	Yes
Industry fixed effects	Yes	Yes	Yes	Yes
Observations	4861	8279	4861	8279
Adj.R-squared	0.220	0.165	0.211	0.163
Empirical *p*-values	0.005 ***		0.037 **	
F-test	25.92 ***		23.29 ***	

Note: *, ** and *** represent significance at the 10%, 5% and 1% levels, respectively, and the *t*-values are in brackets; Empirical *p*-values are used to test the significance of coefficient differences, which are obtained using Bootstrap with 1000 repeated samples; F test is used to examine the coefficient difference between groups as well.

**Table 11:** Moderation tests: the characteristics of industry.

Variables	Heavy	Non-Heavy	Heavy	Non-Heavy
	(1)	(2)	(3)	(4)
	*lnAGreen*	*lnAGreen*	*lnGGreen*	*lnGGreen*
*Digital*	0.407 ***	0.067 *	0.236 **	0.025
	(3.400)	(1.939)	(2.404)	(0.876)
*Size*	0.190 ***	0.168 ***	0.156 ***	0.133 ***
	(11.119)	(18.875)	(11.120)	(18.113)
*Roa*	−0.343	1.212 ***	−0.535 *	0.548 ***
	(−1.003)	(7.156)	(−1.909)	(3.908)
*Lev*	−0.160	0.278 ***	−0.269 ***	0.215 ***
	(−1.634)	(5.239)	(−3.357)	(4.894)
*Growth*	−0.061 *	−0.060 ***	−0.063 **	−0.041 ***
	(−1.763)	(−3.403)	(−2.209)	(−2.831)
*Cfo*	0.561 **	−0.091	0.476 **	0.055
	(2.265)	(−0.707)	(2.345)	(0.521)
*TobinQ*	0.048 ***	−0.005	0.034 **	−0.009
	(2.858)	(−0.643)	(2.416)	(−1.526)
*Dual*	0.002	0.063 ***	0.006	0.041 ***
	(0.045)	(3.534)	(0.188)	(2.750)
*Top1*	0.003 ***	−0.002 ***	0.003 ***	−0.001 **
	(2.804)	(−3.074)	(3.102)	(−2.407)
*Board*	0.550 ***	−0.033	0.555 ***	−0.016
	(6.194)	(−0.666)	(7.622)	(−0.391)
*Indep*	0.731 **	−0.200	1.509 ***	−0.057
	(2.135)	(−1.135)	(5.368)	(−0.391)
*HHI*	−0.980 ***	−0.549 ***	−0.684 ***	−0.378 ***
	(−5.415)	(−9.807)	(−4.609)	(−8.149)
*lnGDP*	0.076 ***	0.105 ***	0.046 ***	0.084 ***
	(3.676)	(8.543)	(2.722)	(8.268)
Constant	−6.100 ***	−4.318 ***	−5.332 ***	−3.521 ***
	(−12.831)	(−16.746)	(−13.666)	(−16.498)
Year fixed effects	Yes	Yes	Yes	Yes
Industry fixed effects	Yes	Yes	Yes	Yes
Observations	2650	10,490	2650	10,490
Adj. R-squared	0.138	0.197	0.140	0.192
Empirical *p*-values	0.012 **		0.007 ***	
F-test	35.03 ***		32.38 ***	

Note: *, ** and *** represent significance at the 10%, 5% and 1% levels, respectively, and the *t*-values are in brackets; Empirical *p*-values are used to test the significance of coefficient differences, which are obtained using Bootstrap with 1000 repeated samples; F test is used to examine the coefficient difference between groups as well.

**Table 12:** Cleaner production effect.

Variables	(1)	(2)
	*GTFP*	*GTFP*
*Digital_it_* × *lnAGreen_i_*_t_	0.020	
	(0.502)	
*Digital_it_* × *lnGGreen_it_*		0.098 **
		(2.052)
*Digital_it_*	−0.046	−0.082 *
	(−0.996)	(−1.772)
*lnAGreen_i_* *_t_*	−0.003	
	(−0.359)	
*lnGGreen_it_*		−0.013
		(−1.477)
*Size*	0.004	0.006
	(0.656)	(0.870)
*Roa*	−0.071	−0.082
	(−0.538)	(−0.622)
*Lev*	−0.050	−0.053
	(−1.369)	(−1.448)
*Growth*	0.046 ***	0.046 ***
	(5.406)	(5.419)
*Cfo*	0.034	0.027
	(0.350)	(0.278)
*TobinQ*	0.004	0.004
	(0.625)	(0.592)
*Dual*	0.001	0.001
	(0.053)	(0.059)
*Top1*	−0.000	−0.000
	(−0.292)	(−0.410)
*Board*	0.031	0.033
	(0.980)	(1.035)
*Indep*	0.298 **	0.315 **
	(2.292)	(2.426)
*HHI*	−0.020	−0.015
	(−0.298)	(−0.225)
*lnGDP*	0.015 *	0.015 *
	(1.763)	(1.795)
Constant	0.629 ***	0.595 ***
	(3.512)	(3.319)
Year fixed effects	Yes	Yes
Industry fixed effects	Yes	Yes
Observations	1390	1390
Adj.R-squared	0.0207	0.0241

Note: *, ** and *** represent significance at the 10%, 5% and 1% levels, respectively, and the *t*-values are in brackets.

**Table 13:** Robustness tests: Heckman two-stage regression.

Variables	Heckman Two-Stage		
	(1)	(2)	(3)
	*Digital_Dummy*	*lnAGreen*	*lnGGreen*
*Digital*		0.107 ***	0.059 **
		(3.205)	(2.121)
*IMR*		0.046	0.193
		(0.233)	(1.186)
*MeanDigital*	2.136 ***		
	(4.472)		
*Size*	−0.032 **	0.166 ***	0.130 ***
	(−2.434)	(19.020)	(18.000)
*Roa*	1.360 ***	0.974 ***	0.525 ***
	(5.334)	(4.335)	(2.825)
*Lev*	0.110	0.194 ***	0.124 ***
	(1.412)	(4.004)	(3.088)
*Growth*	0.002	−0.056 ***	−0.040 ***
	(0.090)	(−3.534)	(−3.105)
*Cfo*	−0.419 **	−0.050	0.020
	(−2.192)	(−0.397)	(0.194)
*TobinQ*	−0.002	−0.003	−0.006
	(−0.225)	(−0.394)	(−1.137)
*Dual*	0.090 ***	0.061 ***	0.050 ***
	(3.347)	(3.134)	(3.101)
*Top1*	−0.000	−0.001 **	−0.001
	(−0.249)	(−1.965)	(−1.301)
*Board*	0.134 *	0.080 *	0.109 ***
	(1.815)	(1.709)	(2.825)
*Indep*	−0.121	0.036	0.269 **
	(−0.465)	(0.228)	(2.052)
*HHI*	0.577 ***	−0.536 ***	−0.318 ***
	(6.163)	(−6.323)	(−4.537)
*lnGDP*	0.114 ***	0.113 ***	0.099 ***
	(6.432)	(6.291)	(6.678)
Constant	−1.579 ***	−4.748 ***	−4.243 ***
	(−4.108)	(−11.147)	(−12.048)
Year fixed effects	Yes	Yes	Yes
Industry fixed effects	Yes	Yes	Yes
Observations	13,140	13,140	13,140
Pseudo/Adj. R-squared	0.0898	0.177	0.172

Note: *, ** and *** represent significance at the 10%, 5% and 1% levels, respectively, and the *t*-values are in brackets.

**Table 14:** The results of covariate adjusting.

Variables	Treatment Group			Control Group (Before)			Control Group (After)		
	Mean	Variance	Skewness	Mean	Variance	Skewness	Mean	Variance	Skewness
*Size*	22.188	1.612	0.765	22.257	1.558	0.735	22.189	1.612	0.765
*Roa*	0.043	0.003	−1.192	0.038	0.003	−1.113	0.043	0.003	−1.192
*Lev*	0.422	0.042	0.201	0.430	0.043	0.210	0.422	0.042	0.201
*Growth*	0.198	0.192	3.777	0.191	0.215	3.644	0.198	0.192	3.777
*Cfo*	0.047	0.005	−0.126	0.047	0.004	−0.045	0.047	0.005	−0.126
*TobinQ*	2.144	1.687	2.337	2.135	1.747	2.358	2.144	1.6873	2.337
*Dual*	0.278	0.201	0.989	0.248	0.186	1.169	0.278	0.201	0.989
*Top1*	35.172	215.873	0.394	35.342	216.655	0.427	35.172	215.873	0.394
*Board*	2.133	0.040	−0.299	2.133	0.041	−0.267	2.133	0.040	−0.299
*Indep*	0.375	0.003	1.307	0.375	0.003	1.344	0.375	0.003	1.307
*HHI*	0.149	0.029	2.880	0.145	0.026	3.097	0.149	0.029	2.880
*lnGDP*	10.494	0.502	−0.553	10.436	0.523	−0.682	10.494	0.502	−0.553

**Table 15:** Robustness tests: entropy balancing matching and independent variable lagged.

Variable	(1)	(2)	(1)	(2)
	*lnAGreen*	*lnGGreen*	*lnAGreen*	*lnGGreen*
*Digital*	0.106 ***	0.055 **		
	(3.74)	(2.00)		
*Digital*_−1_			0.131 ***	0.080 **
			(3.470)	(2.538)
*Size*	0.171 ***	0.134 ***	0.176 ***	0.141 ***
	(17.67)	(20.62)	(19.724)	(19.120)
*Roa*	0.894 ***	0.363 ***	0.812 ***	0.285 **
	(6.13)	(2.88)	(4.826)	(2.046)
*Lev*	0.186 ***	0.111 ***	0.190 ***	0.103 **
	(4.14)	(2.88)	(3.497)	(2.299)
*Growth*	−0.050 ***	−0.040 ***	−0.035	−0.018
	(−3.45)	(−3.11)	(−1.520)	(−0.967)
*Cfo*	−0.019	0.070	0.079	0.139
	(−0.18)	(0.75)	(0.587)	(1.248)
*TobinQ*	−0.003	−0.006	0.003	−0.002
	(−0.45)	(−1.09)	(0.381)	(−0.282)
*Dual*	0.056 ***	0.039 ***	0.063 ***	0.046 ***
	(3.40)	(2.94)	(3.404)	(2.971)
*Top1*	−0.001 **	−0.000	−0.001	−0.000
	(−2.08)	(−1.22)	(−1.352)	(−0.884)
*Board*	0.082 *	0.094 ***	0.095 *	0.125 ***
	(1.66)	(2.58)	(1.887)	(3.002)
*Indep*	0.058	0.285 **	0.044	0.255 *
	(0.34)	(2.19)	(0.246)	(1.702)
*HHI*	−0.570 ***	−0.383 ***	−0.622 ***	−0.407 ***
	(−11.99)	(−8.83)	(−10.157)	(−8.033)
*lnGDP*	0.111 ***	0.085 ***	0.116 ***	0.087 ***
	(11.06)	(9.75)	(9.524)	(8.660)
Constant	−4.744 ***	−3.891 ***	−5.285 ***	−4.389 ***
	(−17.76)	(−20.55)	(−21.641)	(−21.535)
Year fixed effects	Yes	Yes	Yes	Yes
Industry fixed effects	Yes	Yes	Yes	Yes
Observations	13,140	13,140	11,166	11,166
Adj. R-squared	0.176	0.170	0.183	0.176

Note: *, ** and *** represent significance at the 10%, 5% and 1% levels, respectively, and the *t*-values are in brackets.

**Table 16:** Robustness tests: alternative measure of independent variable.

Variable	(1)	(2)	(3)	(4)
	*lnAGreen*	*lnGGreen*	*lnAGreen*	*lnGGreen*
*Digital1*	0.093 ***	0.063 ***		
	(13.816)	(11.190)		
*Digital2*			0.125 ***	0.071 ***
			(14.099)	(9.605)
*Size*	0.174 ***	0.144 ***	0.179 ***	0.147 ***
	(27.798)	(27.648)	(28.643)	(28.401)
*Roa*	0.921 ***	0.391 ***	0.955 ***	0.418 ***
	(7.312)	(3.730)	(7.590)	(3.992)
*Lev*	0.268 ***	0.170 ***	0.240 ***	0.150 ***
	(7.104)	(5.445)	(6.402)	(4.793)
*Growth*	−0.072 ***	−0.059 ***	−0.069 ***	−0.056 ***
	(−5.367)	(−5.233)	(−5.139)	(−4.994)
*Cfo*	0.022	0.060	−0.001	0.039
	(0.233)	(0.777)	(−0.013)	(0.510)
*TobinQ*	−0.002	−0.006	−0.005	−0.008 *
	(−0.450)	(−1.484)	(−1.104)	(−1.938)
*Dual*	0.016	0.021 *	0.022 *	0.026 **
	(1.221)	(1.928)	(1.675)	(2.374)
*Top1*	−0.001 ***	−0.001 **	−0.001 ***	−0.001 **
	(−3.270)	(−2.421)	(−3.156)	(−2.362)
*Board*	0.074 **	0.084 ***	0.077 **	0.085 ***
	(2.023)	(2.756)	(2.122)	(2.795)
*Indep*	0.167	0.310 ***	0.167	0.311 ***
	(1.303)	(2.911)	(1.300)	(2.915)
*HHI*	−0.380 ***	−0.263 ***	−0.380 ***	−0.267 ***
	(−8.399)	(−7.000)	(−8.394)	(−7.099)
*lnGDP*	0.093 ***	0.077 ***	0.094 ***	0.078 ***
	(10.791)	(10.729)	(10.838)	(10.901)
Constant	−4.947 ***	−4.178 ***	−4.884 ***	−4.149 ***
	(−26.817)	(−27.256)	(−26.456)	(−27.023)
Year fixed effects s	Yes	Yes	Yes	Yes
Industry fixed effect	Yes	Yes	Yes	Yes
Observations	19,750	19,750	19,750	19,750
Adj. R-squared	0.191	0.186	0.192	0.184

Note: *, ** and *** represent significance at the 10%, 5% and 1% levels, respectively, and the *t*-values are in brackets.

**Table 17:** Robustness tests: alternative empirical specification.

Variable	Tobit Model		Year × Industry Fixed Effects	
	(1)	(2)	(3)	(4)
	*lnAGreen*	*lnGGreen*	*lnAGreen*	*lnGGreen*
*Digital*	0.106 ***	0.055 **	0.113 ***	0.063 **
	(3.204)	(2.000)	(3.381)	(2.271)
*Size*	0.167 ***	0.134 ***	0.171 ***	0.136 ***
	(21.323)	(20.651)	(21.595)	(20.783)
*Roa*	0.935 ***	0.363 ***	0.928 ***	0.360 ***
	(6.158)	(2.888)	(6.040)	(2.837)
*Lev*	0.191 ***	0.111 ***	0.185 ***	0.110 ***
	(4.091)	(2.883)	(3.931)	(2.819)
*Growth*	−0.056 ***	−0.040 ***	−0.055 ***	−0.040 ***
	(−3.541)	(−3.117)	(−3.452)	(−3.015)
*Cfo*	−0.038	0.070	−0.045	0.066
	(−0.331)	(0.747)	(−0.393)	(0.691)
*TobinQ*	−0.002	−0.006	−0.000	−0.004
	(−0.386)	(−1.094)	(−0.016)	(−0.735)
*Dual*	0.058 ***	0.039 ***	0.061 ***	0.040 ***
	(3.619)	(2.942)	(3.772)	(3.021)
*Top1*	−0.001 *	−0.000	−0.001 **	−0.001
	(−1.956)	(−1.217)	(−2.105)	(−1.427)
*Board*	0.076 *	0.094 ***	0.083 *	0.100 ***
	(1.732)	(2.582)	(1.886)	(2.748)
*Indep*	0.040	0.285 **	0.054	0.308 **
	(0.254)	(2.193)	(0.344)	(2.358)
*HHI*	−0.552 ***	−0.383 ***	−0.601 ***	−0.422 ***
	(−10.520)	(−8.841)	(−10.471)	(−8.883)
*lnGDP*	0.110 ***	0.085 ***	0.109 ***	0.085 ***
	(10.419)	(9.763)	(10.295)	(9.703)
Constant	−4.665 ***	−3.891 ***	−4.699 ***	−3.892 ***
	(−20.399)	(−20.580)	(−20.499)	(−20.529)
Year fixed effects × Industry fixed effects	No	No	Yes	Yes
Year fixed effects	Yes	Yes	No	No
Industry fixed effects	Yes	Yes	No	No
Observations	13,140	13,140	13,140	13,140
Pseudo/Adj.R-squared	0.0766	0.0880	0.172	0.167

Note: *, ** and *** represent significance at the 10%, 5% and 1% levels, respectively, and the *t*-values are in brackets.

## Data Availability

Publicly available datasets were analyzed in this study. This data can be found at: https://www.gtarsc.com/; https://www.cnrds.com/; http://cnki.nbsti.net/.

## References

[1] Cheng Z., Li L., Liu J. (2020). Natural resource abundance, resource industry dependence and economic green growth in China. Resour. Policy.

[2] Wu F., Li W. (2022). Tax incentives and corporate green transformation-empirical evidence based on text recognition of listed companies’ annual reports. Public Financ. Res..

[3] Miao C., Fang D., Sun L., Luo Q. (2017). Natural resources utilization efficiency under the influence of green technological innovation. Resour. Conserv. Recycl..

[4] Huang J.-W., Li Y.-H. (2017). Green innovation and performance: The view of organizational capability and social reciprocity. J. Bus. Ethics.

[5] Amore M.D., Bennedsen M. (2016). Corporate governance and green innovation. J. Environ. Econ. Manag..

[6] Jia P., Li K., Shao S. (2018). Choice of technological change for China’s low-carbon development: Evidence from three urban agglomerations. J. Environ. Manag..

[7] Chanias S., Myers M.D., Hess T. (2019). Digital transformation strategy making in pre-digital organizations: The case of a financial services provider. J. Strateg. Inf. Syst..

[8] Vial G. (2019). Understanding digital transformation: A review and a research agenda. J. Strateg. Inf. Syst..

[9] Yermack D. (2017). Corporate governance and blockchains. Rev. Financ..

[10] Meng S., Wang P., Yu J. (2022). Going abroad and going green: The effects of top management teams’ overseas experience on green innovation in the digital era. Int. J. Environ. Res. Public Health.

[11] Popa S., Soto-Acosta P., Perez-Gonzalez D. (2016). An investigation of the effect of electronic business on financial performance of Spanish manufacturing SMEs. Technol. Forecast. Soc. Chang..

[12] Brenner B. (2018). Transformative sustainable business models in the light of the digital imperative—A global business economics perspective. Sustainability.

[13] Zhang M.J., Lado A.A. (2001). Information systems and competitive advantage: A competency-based view. Technovation.

[14] Frynas J.G., Mol M.J., Mellahi K. (2018). Management innovation made in China: Haier’s Rendanheyi. Calif. Manag. Rev..

[15] Ranta V., Aarikka-Stenroos L., Väisänen J.-M. (2021). Digital technologies catalyzing business model innovation for circular economy—Multiple case study. Resour. Conserv. Recycl..

[16] Costa J., Matias J.C.O. (2020). Open innovation 4.0 as an enhancer of sustainable innovation ecosystems. Sustainability.

[17] Cheng C., Ren X., Dong K., Dong X., Wang Z. (2021). How does technological innovation mitigate CO2 emissions in OECD countries? Heterogeneous analysis using panel quantile regression. J. Environ. Manag..

[18] Lin B.Q., Zhou Y.C. (2021). Does the Internet development affect energy and carbon emission performance?. Sustain. Prod. Consump..

[19] Aaron P., Jason S. (2016). The End of Ownership: Personal Property in the Digital Economy.

[20] Wang C., Liu T., Zhu Y., Lin M., Chang W., Wang X., Li D., Wang H., Yoo J. (2022). Digital economy, environmental regulation and corporate green technology innovation: Evidence from China. Int. J. Environ. Res. Public Health.

[21] Shin D.H., Choi M.J. (2015). Ecological views of big data: Perspectives and issues. Telemat. Inform..

[22] Leong C., Pan S.L., Newell S., Cui L. (2016). The emergence of self-organizing e-commerce ecosystems in remote villages of China: A tale of digital empowerment for rural development. MIS Q..

[23] Hilty L.M., Aebischer B. (2015). ICT for sustainability: An emerging research field. ICT Innov. Sustain..

[24] Siachou E., Vrontis D., Trichina E. (2021). Can traditional organizations be digitally transformed by themselves? The moderating role of absorptive capacity and strategic interdependence. J. Bus. Res..

[25] Zekhnini K., Cherrafi A., Bouhaddou I., Chaouni Benabdellah A., Bag S. (2022). A model integrating lean and green practices for viable, sustainable, and digital supply chain performance. Int. J. Prod. Res..

[26] El-Kassar A.N., Singh S.K. (2019). Green innovation and organizational performance: The influence of big data and the moderating role of management commitment and HR practices. Technol. Forecast. Soc. Chang..

[27] Mubarak M.F., Tiwari S., Petraite M., Mubarik M., Raja Mohd Rasi R.Z. (2021). How industry 4.0 technologies and open innovation can improve green innovation performance?. Manag. Environ. Qual..

[28] Li X., Liu J., Ni P. (2021). The impact of the digital economy on CO2 emissions: A theoretical and empirical analysis. Sustainability.

[29] Ghasemaghaei M., Calic G. (2020). Assessing the impact of big data on firm innovation performance: Big data is not always better data. J. Bus. Res..

[30] Brandt L., Li H. (2003). Bank discrimination in transition economies: Ideology, information or incentives?. J. Comp. Econ..

[31] Goldfarb A., Tucker C. (2019). Digital Economics. J. Econ. Lit..

[32] Cenamor J., Parida V., Wincent J. (2019). How entrepreneurial SMEs compete through digital platforms: The roles of digital platform capability, network capability and ambidexterity. J. Bus. Res..

[33] Shaoxiang J., Yafei L., Nicole Y. (2023). Corporate digitalization, application modes, and green growth: Evidence from the innovation of Chinese listed companies. Front. Environ. Sci..

[34] Li K., Lin B. (2016). Impact of energy technology patents in China: Evidence from a panel cointegration and error correction model. Energy Policy.

[35] Yang G., Cheng S., Gui Q., Chen X. (2022). The coupling and coordination characteristics and influencing factors of green innovation efficiency (GIE) and economic development levels in China. Sustainability.

[36] Mirata M., Emtairah T. (2005). Industrial symbiosis networks and the contribution to environmental innovation: The case of the Landskrona industrial symbiosis programme. J. Clean. Prod..

[37] Matray A. (2021). The local innovation spillovers of listed firms. J. Financ. Econ..

[38] Clarke R.A., Stavins R.N., Greeno J.L., Bavaria J.L., Cairncross F., Esty D.C., Smart B., Piet J., Wells R.P., Gray R. (1994). The challenge of going green. Harv. Bus. Rev..

[39] Huang Z., Liao G., Li Z. (2019). Loaning scale and government subsidy for promoting green innovation. Technol. Forecast. Soc. Chang..

[40] Kong T., Sun R., Sun G., Song Y. (2022). Effects of digital finance on green innovation considering information asymmetry: An empirical study based on Chinese listed firms. Emerg. Mark. Financ. Trade.

[41] Berrone P., Fosfuri A., Gelabert L., Gomez M.L.R. (2013). Necessity as the mother of “green” inventions: Institutional pressures and environmental innovations. Strateg. Manag. J..

[42] Amihud Y., Lev B. (1981). Risk reduction as a managerial motive for conglomerate mergers. J. Econ..

[43] Jin W., Zhang H.Q., Liu S.S., Zhang H.B. (2019). Technological innovation, environmental regulation, and green total factor efficiency of industrial water resources. J. Clean. Prod..

[44] Montalvo C. (2008). General wisdom concerning the factors affecting the adoption of cleaner technologies: A survey 1990–2007. J. Clean. Prod..

[45] Urbinati A., Chiaroni D., Chiesa V., Frattini F. (2020). The role of digital technologies in open innovation processes: An exploratory multiple case study analysis. R D Manag..

[46] Xu E., Yang H., Quan J., Lu Y. (2015). Organizational slack and corporate social performance: Empirical evidence from China’s public firms. Asia Pac. J. Manag..

[47] Li J., Tang Y. (2010). CEO hubris and firm risk taking in China: The moderating role of managerial discretion. Acad. Manag. J..

[48] Simon M., Houghton S.M. (2003). The relationship between overconfidence and the introduction of risky products: Evidence from a field study. Acad. Manag. J..

[49] Lafarre A., Christoph V. (2018). Blockchain technology for corporate governance and shareholder activism. Eur. Corp. Gov. Inst. (ECGI)-Law Work. Pap..

[50] Luong H., Moshirian F., Nguyen L., Tian X., Zhang B. (2017). How do foreign institutional investors enhance firm innovation?. J. Financ. Quant. Anal..

[51] Tian X., Wang T.Y. (2014). Tolerance for failure and corporate innovation. Rev. Financ. Stud..

[52] Chemmanur T.J., Loutskina E., Tian X. (2014). Corporate venture capital, value creation, and innovation. Rev. Financ. Stud..

[53] Kriechel B., Ziesemer T. (2009). The environmental Porter hypothesis: Theory, evidence, and a model of timing of adoption. Econ. Innov. New Technol..

[54] Brnnlund R., Fre R., Grosskopf S. (1995). Environmental regulation and profitability: An application to sweish pulp and paper mills. Environ. Resour. Econ..

[55] Lerner J. (2009). The empirical impact of intellectual property rights on innovation: Puzzles and clues. Am. Econ. Rev..

[56] Maskus K.E., Milani S., Neumann R. (2019). The impact of patent protection and financial development on industrial R&D. Res. Policy.

[57] Lemley M.A., Shapiro C. (2005). Probabilistic patents. J. Econ. Perspect..

[58] Kuo L., Yeh C.-C., Yu H.-C. (2012). Disclosure of corporate social responsibility and environmental management: Evidence from China. Corp. Soc. Responsib. Environ. Manag..

[59] Zhang C., Zhou B., Tian X. (2022). Political connections and green innovation: The role of a corporate entrepreneurship strategy in state-owned enterprises. J. Bus. Res..

[60] Hudson B.A. (2008). Against all odds: A consideration of core-stigmatized organizations. Acad. Manag. Rev..

[61] Chen X., Yi N., Zhang L., Li D. (2018). Does institutional pressure foster corporate green innovation? Evidence from China’s top 100 companies. J. Clean. Prod..

[62] Fernández Y.F., López M.F., Olmedillas B. (2018). Innovation for sustainability: The impact of R&D spending on CO2 emissions. J. Clean. Prod..

[63] Ganda F. (2019). The impact of innovation and technology investments on carbon emissions in selected organization for economic co-operation and development countries. J. Clean. Prod..

[64] Liu Y., Li Q., Zhang Z. (2022). Do smart cities restrict the carbon emission intensity of enterprises? Evidence from a quasi-natural experiment in China. Energies.

[65] Brunnermeier S.B., Cohen M.A. (2003). Determinants of environmental innovation in US manufacturing industries. J. Environ. Econ. Manag..

[66] Hirshleifer D., Hsu P.H., Li D. (2013). Innovative efficiency and stock returns. J. Financ. Econ..

[67] Hadlock C.J., Pierce J.R. (2010). New evidence on measuring financial constraints: Moving beyond the KZ index. Rev. Financ. Stud..

[68] Koerniadi H., Krishnamurti C., Tourani-Rad A. (2014). Corporate governance and risk-taking in New Zealand. Aust. J. Manag..

[69] Tone K. (2001). A slacks-based measure of efficiency in data envelopment analysis. Eur. J. Oper. Res..

[70] Xue Y., Jiang C., Guo Y., Liu J., Wu H., Hao Y. (2022). Corporate social responsibility and high-quality development: Do green Innovation, environmental investment and corporate governance matter?. Emerg. Mark. Financ. Trade.

[71] MacKinnon D.P., Fairchild A.J. (2009). Current directions in mediation analysis. Curr. Dir. Psychol..

[72] Heckman J.J. (1979). Sample selection bias as a specification error. Econometrica.

[73] Xu N., Li X., Yuan Q., Chan K.C. (2014). Excess perks and stock price crash risk: Evidence from China. J. Corp. Financ..

[74] Hainmueller J. (2012). Entropy balancing for causal effects: A multivariate reweighting method to produce balanced samples in observational studies. Polit. Anal..

[75] Moser P., Voena A. (2012). Compulsory licensing: Evidence from the trading with the enemy act. Am. Econ. Rev..

